# Respiratory Tract Viral Infections and Coinfections Identified by Anyplex™ II RV16 Detection Kit in Pediatric Patients at a Riyadh Tertiary Care Hospital

**DOI:** 10.1155/2017/1928795

**Published:** 2017-11-21

**Authors:** Saleh A. Eifan, Atif Hanif, Sameera Mohammed AlJohani, Muhammad Atif

**Affiliations:** ^1^Botany and Microbiology Department, Faculty of Science, King Saud University, Riyadh, Saudi Arabia; ^2^Division of Microbiology, Pathology and Laboratory Medicine, King Abdul-Aziz Medical City, Riyadh, Saudi Arabia; ^3^Department of Physics and Astronomy, King Saud University, Riyadh, Saudi Arabia; ^4^National Institute of Laser and Optronics, Nilore, Islamabad, Pakistan

## Abstract

Respiratory infections are caused by an array of viruses, and limited information is available about viral coexistence, comparative symptoms, and the burden of illness. This retrospective cohort study aimed to determine the etiological agents responsible for respiratory tract infections by Anyplex II RV16 detection kit (RV16, Seegene), involving 2266 pediatric patients with respiratory infections admitted to the Department of Pediatrics at King Abdul-Aziz Medical City, Ministry of National Guard, Riyadh, from July 2014 to June 2015. The most frequent respiratory infections were recorded in the 1 to 5 year age group (44.7%). Rhinovirus (32.5%), Adenovirus (16.9%), and Respiratory syncytial virus (RSV) B (10.4%) were most common. In single viral infections, Rhinovirus (41.2%), Metapneumovirus (15.3%), and Bocavirus (13.7%) were most frequent. In multiple viral infections, Rhinovirus (36.7%), Adenovirus (35.2%), Bocavirus (11.2), RSV B (7.8%), and RSV A (6.7%) were most frequent. No significant difference was observed in clinical presentations; however, rhinorrhea and hypodynamia were significantly associated with viral respiratory infections. Most respiratory viral pathogens peaked during December, January, March, and April. Rhinovirus, Adenovirus, and Bocavirus circulations were detected throughout the year. Winter peaks were recorded for Rhinovirus, RSV B, Adenovirus, and RSV A, whereas the Metapneumovirus, and the Bocavirus peaked in March and April. These findings enhance understanding of viral etiology and distribution to improve respiratory infection management and treatment.

## 1. Introduction

Respiratory tract infections lead to mortality and morbidity in children especially during early years. Among children, more than 80% of respiratory infections are associated with different viral infectious agents. Respiratory virus infections are a major public health problem, due to the ease of spread and considerable morbidity and mortality. The association between respiratory tract infections and different viral pathogens has been reported to vary between 40% and 90% [1–5] globally.

Different studies reported the detection of viruses like human respiratory syncytial virus A (RSV A), human respiratory syncytial virus B (RSV B), human adenovirus (AdV),* Human metapneumovirus * (HMPV), human coronavirus, and human parainfluenza virus (PIV). Children under the age of 5 years were detected with human coronavirus 229E (HCoV-229E), human coronavirus NL63 (HCoV-NL63), human coronavirus OC43 (HCoV-OC43), human parainfluenza virus 1 (PIV-1), human parainfluenza virus 2 (PIV-2), human parainfluenza virus 3 (PIV-3), human parainfluenza virus 4 (PIV-4), human rhinovirus (HRV), human enterovirus (HEV), and human bocavirus (HBoV). Coinfections with different multiple viruses were reported in 15% to 61% of patients. [6–9]. Molecular techniques such as multiplex polymerase chain reaction (PCR) are widely used for the detection and identification of respiratory viruses [10–12] and are helpful in the management and treatment of respiratory infections [13]. Diagnosing respiratory viruses by isolation in cell cultures and serology is time consuming, laborious, expensive, and less sensitive in some cases. Molecular techniques provide quick results with high sensitivity and specificity. Multiplex PCR has been reported as a fast and sensitive assay for respiratory infection detection. Anyplex II RV16 (Seegene, Korea) is a multiplex real-time PCR based kit with Tagging Oligonucleotide Cleavage Extension (TOCE) technology. The pitcher and catcher are two novel components used in TOCE assay for unique signal generation in real time. In TOCE assay detection point is moved from the target sequence to the catcher so it provides the predictable melting temperature analysis for catcher duplex. This process offers the multiplex real-time PCR capability to Anyplex II RV16 kit. [14–17].

Respiratory infections are mostly reported in children living in developing countries. The spread of respiratory infections varies between populations and countries, depending on differences in geography, climate, and socioeconomic conditions [18–21]. The central region (Riyadh region) of Saudi Arabia has a dense population of locals and immigrants whose interaction can affect the transmission patterns of different respiratory viruses. Previous studies have reported the prevalence of a small number of respiratory viruses within different regions of Saudi Arabia, and limited information is available on the seasonal distribution of viruses [22–25]. A better understanding of the local epidemiology and risk factors is critical for the prevention and control of respiratory infections.

This study aimed to determine the distribution of 16 different viruses causing respiratory infections in children, by using RV16, and to compare data on demographic characteristics, symptoms, and single infections or coinfections.

## 2. Methods

### 2.1. Patients and Study Design

This retrospective cohort study included 2266 patients within an age range of 0 to 14 years from July 2014 to June 2015 with suspected acute respiratory illness and respiratory infection. The patients were examined clinically and initially diagnosed by an admitting physician. Nasopharyngeal aspirates, bronchoalveolar lavages, and nasopharyngeal swab specimens were sent for PCR analysis of respiratory viruses to the Division of Microbiology, Pathology and Laboratory Medicine, King Abdul-Aziz Medical City, Riyadh, Saudi Arabia. When more than one virus was detected simultaneously from single or multiple samples of the same patient, the findings were recorded as multiple infections. Patient demographic information was obtained from medical records. Demographic and clinical data were recorded in a standardized Performa, including age, sex, and clinical presentation. The study was approved by the Ethics Committee at King Abdul-Aziz Medical City.

### 2.2. Patient Inclusion and Exclusion Criteria

Respiratory virus samples were collected from patients presenting to hospital for the first time with symptoms of respiratory infection or within 7 days of admission. The time frame included for the sampling criteria was based on the incubation periods of these viruses [26, 27].

Patient samples collected after 7 days from the date of admission were excluded from the study, as these samples were considered nosocomial infections.

### 2.3. PCR

Nucleic acids were extracted from all samples using Microlab Nimbus IVD (Seegene Inc.), and RNAs were used for cDNA synthesis using cDNA Synthesis Premix (Seegene Inc.). The samples were tested by using Anyplex II RV16 detection kit (Seegene Inc.) according to the manufacturer's instructions. The assay was used to detect Flu-A, Flu-B, RSV A, RSVB, AdV, HMPV, HCoV-229E, HCoV-NL63, HCoV-OC43, PIV-1, PIV-2, PIV-3, PIV-4, HRV, HEV, and HBoV. Reaction mixtures for virus detection were divided into two panels: A and B. Each panel was used to detect 8 viruses with appropriate controls. Two types of DNA and 14 types of RNA viruses were amplified and detected by using CFX 96 Real-Time PCR Thermal cycler (Bio-Rad). Seegene Viewer software was used to analyze the amplification results. The study was approved by the Research and Ethical Committee of King Abdul-Aziz Medical City, Riyadh.

### 2.4. Statistical Analysis

Data analysis was performed by using SPSS (version 22.0; IBM). Differences in the distribution of categorical variables were compared using chi-square or Fisher's exact tests. A* P v*alue of ≤0.05 was considered significant.

## 3. Results

From among 2266 hospitalized patients, different respiratory infectious viruses were detected in 2041 (91.6%) samples (1082 male and 959 female participants). Among age group of 1 to 5 years 44.7% respiratory infections were recorded and 7.8% infections were detected in age group of 11 to 14 years (Table 1).

The respiratory viruses detected were HRV in 664 samples (32.5%), AdV in 344 samples (16.9%), RSV B in 212 samples (10.4%), HBoV in 171 samples (8.4%), RSV A in 124 samples (6.1%), HMPV in 99 samples (4.9%), Flu-A in 95 samples (4.7%), HEV in 92 samples (4.5%), PIV-4 in 74 samples (3.6%), Flu- B in 51 samples (2.5%), HCoV-OC43 in 50 samples (2.4%), PIV-3 in 31 samples (1.5%), HCoV-NL63 in 12 samples (0.6%), PIV-2 in 8 samples (0.4%), PIV-1 in 7 samples (0.3%), and HCoV-229E in 7 samples (0.3%). A total of 863 (42.7%) samples were found to be infected with multiple viral infections. The AdV group depicted 29.4% multiple viral infections, whereas the PIV-1 virus group showed 0.1% multiple viral infections (Table 2).

**Figure 1:**
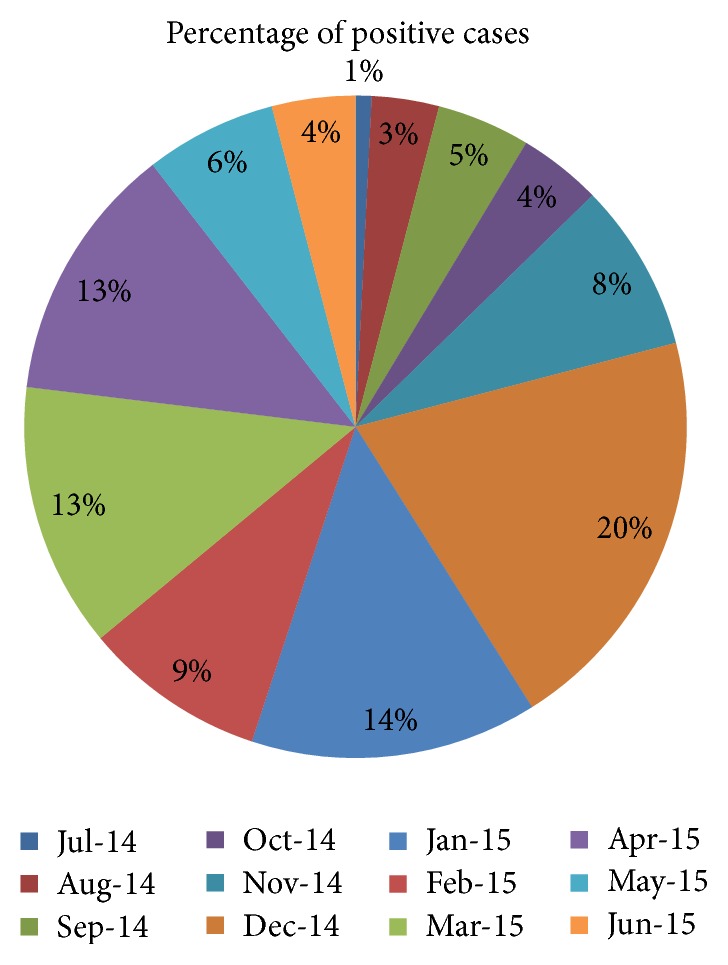
Monthly distribution of respiratory viral infections from July 2014 to June 2015.

**Figure 2:**
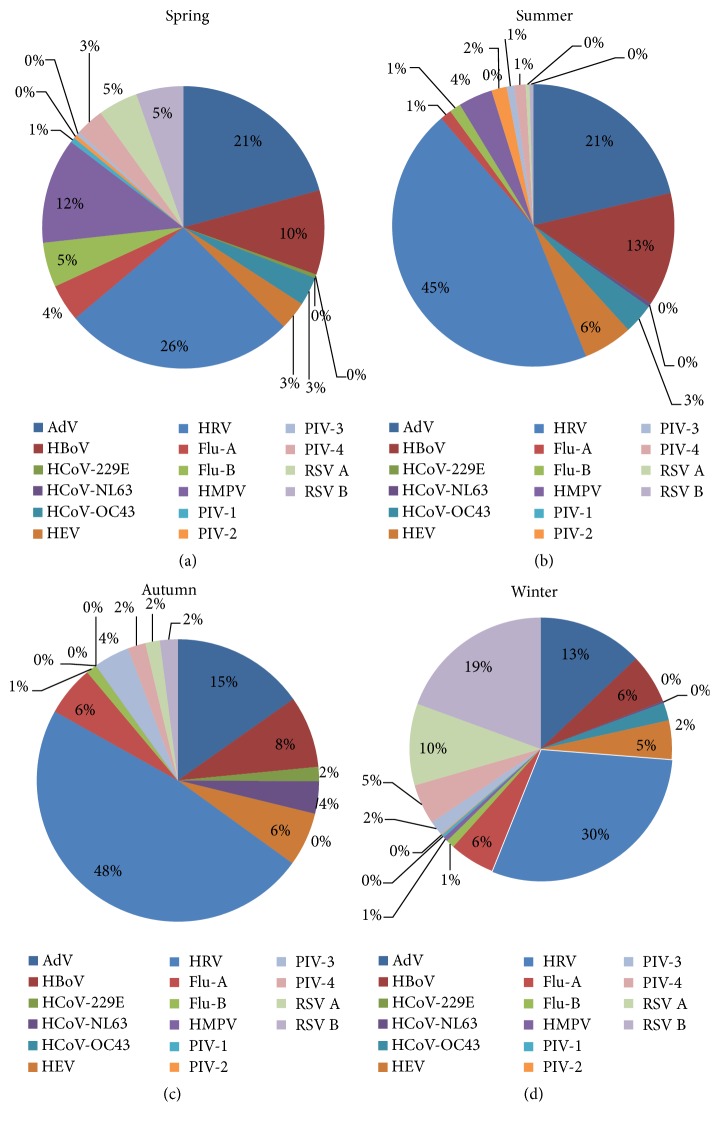
Distribution of sixteen respiratory viruses during spring, summer, autumn, and winter.

**Figure 3:**
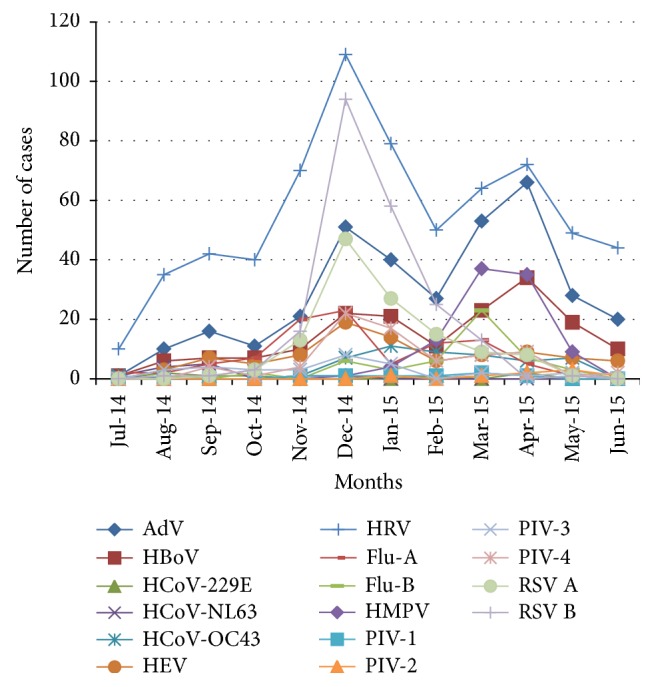
Cocirculation patterns of respiratory viruses.

Statistically significant relationships were found between the detection of single and multiple virus infections in the AdV and HEV groups (*P* < 0.05). In multiple viral infections coinfections were recorded among HRV (36.7%), AdV (35.2%), HBoV (11.2), RSV B (7.8%), and RSV (6.7%), respectively (Table 3). Patients' clinical presentations with different etiological agents were compared and listed in Table 4. The monthly distribution patterns of different respiratory viruses were shown in Figure 1. The prevalence rate of sixteen respiratory viruses during spring, summer, autumn, and winter was 34.4%, 11.2%, 11.9%, and 42.3%, respectively (Figure 2). Seasonal distributions of different respiratory viruses were shown in Figure 3.

## 4. Discussion

In this study, respiratory infectious viruses were recorded in 2041 out of 2266 (91.6%) samples. In comparison to other studies, a higher infection percentage was detected among pediatric patients: Saudi Arabia (12 viruses, 109 out of 135, 80.7%) [25], Honduras (16 viruses, 260 out of 345, 75.4%,) [28], China (16 viruses, 248 out of 490, 50.6%,) [19], and Greece (17 viruses, 428 out of 611, 70.0%,) [29]. Our findings are in line with previous studies that reported viral detection rates ranging between 47 and 95% [1, 3, 7, 30]. The wide ranging differences in viral pathogen detection rates may be attributed to heterogeneity within the study population, genetic variability, the types and numbers of viral pathogens included for testing, and the methods used for testing [1, 20, 31].

Most of the respiratory infections were recorded in the 1-to-5-year-old age group, followed by the age group >1 year old (Table 1), whereas a previous study [25] detected a higher ratio of respiratory pathogens among children less than 1 year of age. The higher rate of infections in infants and young children may be related to underdeveloped or weak immune systems, less healthy living conditions, greater pathogen exposure, and poorer hygiene [1, 4, 10, 31].

The detection of disease-causing viral pathogen plays an important role in patient management and treatment. In this study, the most common respiratory viruses detected were HRV (32.5%), AdV (16.9%), and RSV B (10.4%). A previous study [25] reported detection rates for RSV (23.9%), HRV (14.7%), and AdV (11%) in Saudi Arabia. In another study in China, influenza viruses (18.50%), RSV (7.86%), and AdV (3.47%) were found to be the most common respiratory pathogens [32].

In multiple viral infections, HRV (36.7%), AdV (35.2%), HBoV (11.2), RSV B (7.8%), and RSV A (6.7%) were found most frequently. In single viral infections, HRV (41.2%), HMPV (15.3%), and HBoV (13.7%) were detected most frequently (Table 3). AdV and HEV were found to be significantly higher in number than other viruses. This finding suggests that the occurrence of these viruses may facilitate other viral pathogens to infect the patient. The mixed viral infection rate was 42.3% for all samples in our study. HRV (36.7%), AdV (35.2%), HBoV (11.2), RSV B (7.8%), and RSV A (6.7%) were found among most frequent coinfected groups. Another study has reported detecting multiple viral infections involving rhinovirus, AdV, and HCoV-OC43 groups [2]. HRV and AdV have also been reported as the leading viral pathogens involved in mixed viral infectious agents among children [33]. These results further suggest that some viral groups facilitate infection or colonization by other viruses in the same patients. Viral coinfection or the detection of two or more viral pathogens in a single patient may be attributed to asymptomatic persistence or the shedding of viruses [3]. The great variations in detection rates of multiple viral infections, in different studies, may be related to differences in study populations, locations, study periods, environmental factors, the number of viral pathogens tested, and differences in diagnostic techniques [2, 8, 34]. Similarities in clinical presentations of patients infected with different respiratory viruses make it difficult to diagnose the precise etiological agent based on clinical signs.

In our study, fever and cough were observed as common symptoms in patients infected with different respiratory viruses, and a strong association of rhinorrhea and hypodynamia was observed alongside other nonsignificant clinical symptoms.

In the present study, RSV A, RSV B, HRV, and HEV showed peaks of activity during December, whereas HMPV and HBoV peaked in March and April. HRV, AdV, and HBoV circulations were observed throughout the year. Winter seasonal peaks were recorded for RSV A and RSV B. An increase in the detection of viral pathogens frequencies occurred during cold and rainy seasons. Seasonal variations of different respiratory viruses have been studied [35] and a significantly higher number of viruses have been detected during winter (54.7%) compared to summer (31%), with HRV being the most common pathogen in all seasons. Other studies have reported detecting most influenza viruses in November and December, and RSV was detected most frequently between December and February [4, 8, 9, 28].

### 4.1. Limitations of Study

This study lacked further information on bacterial cultures taken from the samples used for viral pathogen detection. Provision of this information would have been helpful for the assessment of concomitant infection by respiratory viruses and bacterial pathogens to help control and treat respiratory infections. This study incorporated data for a period of one year only, but the inclusion of data from the previous and following years could have provided more information on circulatory patterns of respiratory viruses. Finally, this study was conducted on a single site in the Riyadh region, and virus spread and circulation patterns are likely to differ in other regions of Saudi Arabia, such as Jeddah and Dammam.

## 5. Conclusions

In conclusion, our study provides information regarding the circulatory patterns and seasonal distribution of human respiratory pathogens in the central region of Saudi Arabia. Rhinoviruses, adenoviruses, and RSV were found to be the most common pathogens in pediatric patients. The RV16 based PCR diagnostic approach increased our understanding of viral etiology for better management, control, and treatment of respiratory infections.

## Figures and Tables

**Table 1:** Distribution of viral detection by age and sex.

	Virus positive	%
*Age (years)*		
<1	717	35.1
1–5	913	44.7
6–10	252	12.3
11–14	159	7.8

*Sex*		
Male	1082	53.0
Female	959	47.0
*P*	0.193	

Data presented as numbers and percentages (%). Significant difference: *P* ≤ 0.05.

**Table 2:** Distribution of single and multiple viral infections.

Virus groups	Total number of positive samples		Number of positive samples with single virus		Number of positive samples with multiple viruses	
AdV	344	(16.9)	90	(7.6)	254	(29.4)
HBoV	171	(8.4)	98	(8.3)	73	(8.5)
HCoV-229E	7	(0.3)	4	(0.3)	3	(0.3)
HCoV-NL63	12	(0.6)	4	(0.3)	8	(0.9)
HCoV-OC43	50	(2.4)	37	(3.1)	13	(1.5)
HEV	92	(4.5)	19	(1.6)	73	(8.5)
HRV	664	(32.5)	411	(34.9)	253	(29.3)
Flu-A	95	(4.7)	76	(6.5)	19	(2.2)
Flu-B	51	(2.5)	41	(3.5)	10	(1.2)
HMPV	99	(4.9)	71	(6.0)	28	(3.2)
PIV-1	7	(0.3)	6	(0.5)	1	(0.1)
PIV-2	8	(0.4)	6	(0.5)	2	(0.2)
PIV-3	31	(1.5)	26	(2.2)	5	(0.6)
PIV-4	74	(3.6)	60	(5.1)	14	(1.6)
RSV A	124	(6.1)	92	(7.8)	32	(3.7)
RSV B	212	(10.4)	137	(11.6)	75	(8.7)

Data presented as numbers and percentages (%).

**Table 3:** Relationship between single and multiple viral infections.

Viral groups	Single viral infections (%)	Multiple viral infections (%)	*P*
AdV	11.3	35.2	<0.05
HBoV	13.7	11.2	0.638
HCoV-229E	1.5	1.3	0.451
HCoV-NL63	1.6	1.9	0.186
HCoV-OC43	7.9	3.2	0.356
HEV	4.9	12.7	<0.05
HRV	41.2	36.7	0.571
Flu-A	9.7	4.5	0.371
Flu-B	7.2	3.4	0.135
HMPV	15.3	8.7	0.147
PIV-1	1.1	0.5	0.119
PIV-2	1.1	0.7	0.157
PIV-3	3.4	1.9	0.213
PIV-4	4.5	2.3	0.183
RSV A	11.3	6.7	0.295
RSV B	12.5	7.8	0.314

Significant difference *P* ≤ 0.05.

**Table 4:** Clinical presentations of the patients.

	Fever (≥38)	Cough	Rhinorrhea	Sore throat	Expectoration	Tachypnea	Dyspnea	Chest pain	Hypodynamia	Bellyache	Diarrhea	Headache
AdV	40	12	13	8	10	9	8	12	4	8	25	1
	(11.6)	(3.5)	(3.8)	(2.3)	(2.9)	(2.6)	(2.3)	(3.5)	(1.2)	(2.3)	(7.3)	(0.3)
HBoV	24	2	17	2	3	4	2	5	1	2	10	3
	(14.0)	(1.2)	(9.9)	(1.2)	(1.8)	(2.3)	(1.2)	(2.9)	(0.6)	(1.2)	(5.8)	(1.8)
HCoV-229E	2	3	2	4	3	2	4	3	2	3	1	3
	(28.6)	(42.9)	(28.6)	(57.1)	(42.9)	(28.6)	(57.1)	(42.9)	(28.6)	(42.9)	(14.3)	(42.9)
HCoV-NL63	3	4	5	2	1	2	3	4	5	4	3	2
	(25.0)	(33.3)	(41.7)	(16.7)	(8.3)	(16.7)	(25.0)	(33.3)	(41.7)	(33.3)	(25.0)	(16.7)
HCoV-OC43	9	2	1	2	2	3	1	2	2	3	1	3
	(18.0)	(4.0)	(2.0)	(4.0)	(4.0)	(6.0)	(2.0)	(4.0)	(4.0)	(6.0)	(2.0)	(6.0)
HEV	41	4	23	7	4	5	3	4	6	3	2	3
	(44.6)	(4.3)	(25.0)	(7.6)	(4.3)	(5.4)	(3.3)	(4.3)	(6.5)	(3.3)	(2.2)	(3.3)
HRV	137	232	249	41	18	21	15	21	23	29	19	42
	(20.6)	(34.9)	(37.5)	(6.2)	(2.7)	(3.2)	(2.3)	(3.2)	(3.5)	(4.4)	(2.9)	(6.3)
Flu-A	31	18	20	26	18	15	13	12	25	21	15	28
	(32.6)	(18.9)	(21.1)	(27.4)	(18.9)	(15.8)	(13.7)	(12.6)	(26.3)	(22.1)	(15.8)	(29.5)
Flu-B	18	9	12	15	9	8	11	10	12	15	10	21
	(35.3)	(17.6)	(23.5)	(29.4)	(17.6)	(15.7)	(21.6)	(19.6)	(23.5)	(29.4)	(19.6)	(41.2)
HMPV	15	3	4	4	3	3	4	2	1	2	1	3
	(15.2)	(3.0)	(4.0)	(4.0)	(3.0)	(3.0)	(4.0)	(2.0)	(1.0)	(2.0)	(1.0)	(3.0)
PIV-1	3	2	1	3	2	3	2	1	2	3	2	1
	(42.9)	(28.6)	(14.3)	(42.9)	(28.6)	(42.9)	(28.6)	(14.3)	(28.6)	(42.9)	(28.6)	(14.3)
PIV-2	2	1	3	1	4	2	1	3	2	1	3	1
	(25.0)	(12.5)	(37.5)	(12.5)	(50.0)	(25.0)	(12.5)	(37.5)	(25.0)	(12.5)	(37.5)	(12.5)
PIV-3	6	2	3	4	2	1	4	2	3	2	1	3
	(19.4)	(6.5)	(9.7)	(12.9)	(6.5)	(3.2)	(12.9)	(6.5)	(9.7)	(6.5)	(3.2)	(9.7)
PIV-4	10	3	4	3	2	4	1	4	3	1	1	2
	(13.5)	(4.1)	(5.4)	(4.1)	(2.7)	(5.4)	(1.4)	(5.4)	(4.1)	(1.4)	(1.4)	(2.7)
RSV A	18	11	13	5	9	16	6	7	3	4	14	5
	(14.5)	(8.9)	(10.5)	(4.0)	(7.3)	(12.9)	(4.8)	(5.6)	(2.4)	(3.2)	(11.3)	(4.0)
RSV B	33	17	24	11	12	20	5	7	3	7	17	13
	(15.6)	(8.0)	(11.3)	(5.2)	(5.7)	(9.4)	(2.4)	(3.3)	(1.4)	(3.3)	(8.0)	(6.1)

*P*	0.09	0.26	< 0.05	0.18	0.13	0.32	0.21	0.15	< 0.05	0.41	0.28	0.35

Data presented as numbers and percentages (%), significant difference *P* ≤ 0.05.
